# Inflammation and neuropsychiatric symptoms in Parkinson's disease: a genetic association

**DOI:** 10.3389/fnagi.2026.1877657

**Published:** 2026-08-31

**Authors:** Alexandra Ribeiro Gonçalves, Alexandre Mendes, Joana Damásio, Nuno Vila-Chã, Daniela Boleixa, Cristina Santos, Bárbara Leal, Sara Cavaco

**Affiliations:** 1 Neuropsychology Service, Centro Hospitalar Universitário de Santo António, Unidade Local de Saúde Santo António, Porto, Portugal; 2 Faculty of Medicine, University of Porto, Porto, Portugal; 3 Neurology Department, Centro Hospitalar Universitário de Santo António, Unidade Local de Saúde Santo António, Porto, Portugal; 4 Unit for Multidisciplinary Research in Biomedicine, School of Medicine and Biomedical Sciences, University of Porto, Porto, Portugal; 5 ITR—Laboratory for Integrative and Translational Research in Population Health, Porto, Portugal; 6 Immunogenetics Laboratory, Molecular Pathology and Immunology Department, School of Medicine and Biomedical Sciences, University of Porto, Porto, Portugal

**Keywords:** hallucinations, impulse control disorder (ICD), interleukin-1β (IL-1β) rs16944, neuropsychiatry, Parkinson's disease

## Abstract

**Background:**

There has been some evidence that neuroinflammation is involved in Parkinson's disease (PD) and in psychiatric disorders.

**Objective:**

To explore the effects of IL-1β rs16944 on PD patients' neuropsychiatric symptoms.

**Methods:**

Two hundred and eighty-one consecutive PD patients underwent a neurological and neuropsychological evaluations, which included: Unified Parkinson's Disease Rating Scale, Schwab and England Activities of Daily Living scale, Questionnaire for Impulsive-Compulsive Disorders in Parkinson's Disease—Anytime Short, Hospital Anxiety and Depression Scale and Dementia Rating Scale-2. The Neuropsychiatric Inventory (NPI) was also applied to patients' caregivers/informants. In addition to IL-1β rs16944, DRD3 rs6280 and GRIN2B rs7301328 were also genotyped. Non-parametric group comparisons and linear and logistic regressions were used for data analyses.

**Results:**

The IL-1β rs16944 (specifically TT vs. CC) genotype was associated with increased frequency of impulse-control behaviors and disorders (ICBDs) and impulse control disorders (ICDs; OR = 3.54, *p* = 0.004 and OR = 3.63, *p* = 0.006, respectively) and with more NPI reports of hallucinations (OR = 3.92, *p* = 0.014) and more neuropsychiatric symptoms (ß = 0.73, *p* = 0.019). The rs16944 T allele was related to more NPI reports of hallucinations (OR = 2.31, *p* = 0.032) and agitation/aggression (OR = 2.02, *p* = 0.039).

**Conclusion:**

Study findings suggest that a polymorphism previously associated with increased IL-1β expression may increase the risk of certain neuropsychiatric symptoms in PD.

## 1 Background

Parkinson's disease (PD) is a common neurodegenerative disease often associated with older age. The clinical presentation includes both classical motor symptoms (i.e., bradykinesia, rigidity, resting tremor, and postural instability) and non-motor symptoms (e.g., neuropsychiatric symptoms, cognitive impairment, anosmia, sleep disturbances, and dysautonomia) (Litvan et al., 2003; Postuma et al., 2015; Sauerbier et al., 2016). The prevalence and progression of neuropsychiatric symptoms in the course of PD are not fully understood; however, they have been associated with increased rates of functional disability and physical and cognitive decline, with a greater risk of dementia, and with reduced quality of life (Burchill et al., 2024; Weintraub et al., 2022). The neuropsychiatric symptoms can be grouped into those related to affect (i.e., depression, and anxiety), those related to perception and thinking (e.g., psychosis), and those related to motivation (i.e., apathy and impulsive compulsive behaviors) (Weintraub et al., 2022). Impulse-control disorders (ICDs) in PD include pathological gambling, compulsive or binge eating, compulsive buying, and hypersexuality. Other commonly related behaviors include punding (i.e., stereotyped behaviors characterized by simple, repetitive, and non-goal-directed activities), hobbyism and dopamine dysregulation syndrome (DDS; i.e., compulsive overuse of PD medication). Several polymorphisms (e.g., DRD3 rs6280, GRIN2B rs7301328) have been implicated in cognitive impairment and neuropsychiatric symptoms in PD (Dulski et al., 2022; Gonçalves et al., 2024; Magistrelli et al., 2021).

In non-PD populations, there is strong evidence that neuroinflammation plays an important role in psychiatric disorders, such as, schizophrenia, bipolar disorder, major depressive disorder, and posttraumatic stress disorder (Cheng et al., 2023; Dunn et al., 2020; González-Blanco et al., 2019; Nascimento et al., 2024). Neuroinflammation is also increasingly recognized as a pathophysiological feature in the early stages of PD (Boyd et al., 2022; Deleidi and Gasser, 2013; Oliveira et al., 2024). It has been suggested that neuroinflammatory mechanisms are involved not only in the cascade of events that promotes neuronal degeneration in PD (Zhang et al., 2023), but also in the development of PD related non-motor symptoms (Lindqvist et al., 2012; Oliveira et al., 2024). Pro-inflammatory cytokines and of other inflammation markers (e.g., interleukin-1β, interleukin-6, interleukin-10, tumor necrosis factor-α, and C-reactive protein) are commonly elevated in PD, in both peripheral blood and cerebrospinal fluid (Qu et al., 2023). Some of these inflammatory factors (e.g., interleukin-6, interleukin-10, tumor necrosis factor-α, and C-reactive protein) are also positively correlated with depression and hallucinations in PD (Sawada et al., 2014; Swann et al., 2024).

Like other proinflammatory cytokines, IL-1β has been found to be increased in the brain, serum, and CSF of patients with psychiatric disorders, such as schizophrenia and bipolar disorder (Cheng et al., 2023; González-Blanco et al., 2019; Wang and Miller, 2018), and in PD, including in the potential prodromal stage (Dzamko, 2023). IL-1β circulating levels has been linked to more severe motor symptoms in PD (Fan et al., 2020). However, no significant association between circulating levels of IL-1β and depression symptoms has been reported in patients with PD (Swann et al., 2024). It's still unclear whether IL-1β is related to other neuropsychiatric symptoms (e.g., hallucinations and impulse control disorders) in PD.

The rs16944 polymorphism is located in the IL-1β promoter region and is known to increase the IL-1β expression (Hall et al., 2004). Some studies suggest that carriers of the homozygous variant genotype of rs16944 (i.e., TT genotype) are at increased risk for PD (McGeer et al., 2002; Wahner et al., 2007), whereas other studies reported no significant association (Chu et al., 2012; Ulhaq and Garcia, 2020; Yi et al., 2023). In non-PD populations, several genetic studies have linked rs16944 TT genotype with increased risk of schizophrenia and bipolar disorder (Papiol et al., 2004; Xu and He, 2010).

The primary aim of this study was to investigate the effects of IL-1β rs16944 on neuropsychiatric symptoms in a cohort of PD patients. As a secondary goal, the study also explored the effects of DRD3 rs6280 and GRIN2B rs7301328 on neuropsychiatric symptoms.

## 2 Methods

### 2.1 Subjects

Our cohort of PD patients was previously described, as part of a comprehensive observational study (Mendes et al., 2015). Briefly, from a series of 322 consecutive non-surgically treated PD subjects, only one patient refused to participate in the study. From this cohort (*n* = 321), six died prior to assessment (i.e., 2 patients from respiratory infection, one from heart disease, and 3 from unknown cause), two were excluded because they developed other debilitating conditions, and 13 moved geographically to a region not dependent from our center or could not be reached between inclusion and assessment. For the present study, 19 patients were *a posteriori* excluded, because genotyping could not be conducted (*n* = 16) or the diagnosis changed during follow-up (*n* = 3). A total of 281 PD participants were included in the study.

All participants (or legal representatives) were informed about the nature of the study and gave their consent for participation, in accordance with the Declaration of Helsinki. The study was approved by local ethics committee.

### 2.2 Procedures

#### 2.2.1 Assessment protocol

Clinical records were reviewed to determine age at disease onset and laterality of motor symptoms at PD onset and to identify current medication. Age at the first motor symptom was considered age at PD onset. Current anti-parkinsonian medication was converted to levodopa equivalent dose (Tomlinson et al., 2010). Two movement disorder specialists (AM, NVC) evaluated patient's motor symptoms, using the Unified Parkinson's Disease Rating Scale (UPDRS) parts II and III (Fahn et al., 1987). The UPDRS-II assessed motor experiences of daily living in OFF and ON states, based on the patient and/or the caregiver/informant report. UPDRS-III was used to evaluate motor symptoms after at least 12 h without anti-parkinsonian medication (OFF state) and after taking their habitual morning dose of anti-parkinsonian medication (ON state). The Schwab and England Activities of Daily Living scale (S&E) (Schwab and England, 1969) in OFF and ON states and the Questionnaire for Impulsive-Compulsive Disorders in Parkinson's Disease—Anytime Short (QUIP-Anytime) (Weintraub et al., 2009) were also applied by the movement disorders specialists based on an interview to the patient and/or the caregiver/informant. The S&E measured patient's ability to perform daily activities. QUIP-Anytime assessed the presence of impulse-control (i.e., compulsive gambling, buying, sexual behavior, and eating behaviors) and related behaviors (hobbyism, punding, and dopamine dysregulation syndrome) disorders (ICBDs) since PD diagnosis.

A trained neuropsychologist, blinded to the motor assessment scores, applied the Portuguese versions of the Neuropsychiatric Inventory (NPI) (Cummings, 1997; Ferreira et al., 2015), the Hospital Anxiety and Depression Scale (HADS) (Pais-Ribeiro et al., 2007; Zigmond and Snaith, 1983), and the Dementia Rating Scale-2 (DRS-2) (Cavaco and Teixeira-Pinto, 2011; Jurica et al., 2001). NPI was completed by caregivers/informants of 191 PD patients. This questionnaire assessed the presence, frequency, and severity of behavioral symptoms in the past 4 weeks and covered 12 behavioral domains, namely, delusions, hallucinations, agitation/aggression, depression/dysphoria, anxiety, elation/euphoria, apathy/indifference, disinhibition, irritability/lability, aberrant motor behavior, sleep and nighttime behavior disorders, and appetite and eating disorders. The NPI total score corresponds to the sum of frequency x severity for all domains. The presence of symptoms on each domain was also explored. HADS, a self-report questionnaire composed by 14-items, explored the presence and severity of anxiety and depression symptoms in the past week. Scores ≥11 on the HADS subscales were indicative of anxiety and depression. DRS-2 was administered to screen global cognitive status. For those medically treated for PD (98% of the patients), HADS and DRS-2 were applied to the patient under the effects of his/her regular anti-parkinsonian medication (ON state). Due to the patient's inability to complete the task, HADS was discontinued in nine patients and DRS-2 was discontinued in two patients.

#### 2.2.2 DNA extraction and genotyping

Peripheral blood samples (10 mL) were collected in 5% EDTA tubes (VACUETTE^®^, Portugal). Genomic DNA was obtained from proteinase-K treated peripheral blood leukocytes with a Salting-Out procedure. The rs16944 (−511 C>T, *IL-1*β), rs6280 (25T>C, *DRD3*), and rs7301328 (366 C>G, *GRIN2B*) polymorphisms were genotyped using pre-designed TaqMan allelic discrimination assays (C_1839943_10, C_949770_20 and C_25472761_30; Thermo Fisher Scientific, USA). The reactions were performed with the NzySpeedy qPCR Master Mix (Nzytech, Portugal) on a Rotor-Gene 6000 Real-Time PCR system (Corbett Life Science, Foster City, CA, USA).

#### 2.2.3 Statistical analyses

Descriptive statistics were used for group characterization. Chi-square test and Mann-Whitney test was used to compare patients with and without NPI filled by a caregiver / informant. Chi-square test (or Fisher's exact test), Mann-Whitney test, and Kruskal-Wallis test were applied to identify associations between IL-1β rs16944, DRD3 rs6280, and GRIN2B rs7301328 genotypes and alleles and measures of neuropsychiatric symptoms (i.e., QUIP-Anytime, NPI, and HADS). The effect sizes were calculated using Phi, Cramer's V, and Eta^2^. The Benjamini-Hochberg procedure was also applied in order to calculate the False Discovery Rate (FDR) for primary analyses (i.e., related IL-1β rs16944) and for the exploratory analyses (i.e., related to DRD3 rs6280 and GRIN2B rs7301328). Significant associations (*p* < 0.05) were further explored with linear and logistic regressions. Other potential predictors (i.e., sex, age, age at disease onset, laterality of motor symptoms at onset, disease duration, levodopa equivalent dose, dopamine agonist, and UPDRS-II, UPDRS-III and S&E in OFF and ON states and the absolute difference between states) of neuropsychiatric symptoms were also analyzed. ON state assessments in unmedicated patients were treated as missing data. Due to the right-skewed distribution of NPI total scores, these were converted into natural logarithmic scale; prior to this transformation, the zero score was replaced by one. Multiple linear and logistic regressions were applied to analyze the genotypic and allelic effects on neuropsychiatric symptoms while taking into consideration other significant predictors (*p* < 0.05) identified in the univariate analyzes. The inclusion of ON state assessment measures (either direct and derived) in the multiple regression model automatically excluded non-medicated patients. So, whenever justified, two multiple regression models were created for a single dependent variable: one without ON state assessment (either direct and derived) and one that included significant ON state measures. A stepwise selection approach was applied to the multiple linear regression analyses (i.e., backward selection) and to the multiple logistic regression analyses (i.e., backward likelihood ratio). For both, the threshold for variable was set at *p* > 0.100. Multicollinearity statistics, namely tolerance and variance inflation factor (VIF), were verified in the final regression models. None of the final regression models had a tolerance value < 0.20 nor a VIF value >3. The number of events per independent predictor was set at approximately 10 or more. Linear and logistic regression assumptions were verified.

## 3 Results

### 3.1 Cohort characterization

All PD patients, but one, were Caucasian (99.6%). The demographic and clinical characteristics of the whole cohort at the time of the assessment are presented in Table 1. PD patients with NPI filled by a caregiver/informant were older, had fewer years of education, had longer disease duration, were taking higher levodopa equivalent dose, had more motor symptoms on the UPDRS-III and poorer cognitive performance on the DRS-2, and were taking less dopamine agonist (Table 1).

**Table 1:** Demographical and clinical characterization of the total sample (*n* = 281) and of patients with and without NPI.

Demographic and clinical variables			Total (*n* = 281)	NPI		
				Without (*n* = 90)	With (*n* = 191)	*p*
Sex		Male	146 (52%)	47 (52%)	99 (52%)	0.951
Age		Years	70 (63, 77)	66 (58, 72)	72 (64, 78)	< 0.001
Education		Years	4 (4, 6)	4 (4, 9)	4 (4, 4)	< 0.001
Age at disease onset		Years	61 (53, 69)	60 (51, 66)	63 (54, 70)	0.032
Laterality		Right	154 (55%)	49 (54%)	105 (55%)	0.934
DD		Years	7 (4, 11)	5 (4, 7)	8 (5, 12)	< 0.001
LED		Mg	760 (460, 1,155)	540 (370, 885)	940 (540, 1,300)	< 0.001
DA^*^		Yes	114 (41%)	46 (51%)	68 (36%)	0.013
UPDRS-II	OFF	Score	14 (9, 21)	10 (7, 13)	16 (11, 23)	< 0.001
	ON^**^	Score	8 (4, 12)	5 (3, 8)	9 (6, 13)	< 0.001
	OFF- ON^**^	Score	6 (3, 9)	4 (2, 7)	7 (4, 10)	< 0.001
UPDRS-III	OFF	Score	32 (25, 40)	25 (20, 32)	35 (28, 43)	< 0.001
	ON^**^	Score	22 (16, 27)	16 (13, 22)	24 (17, 30)	< 0.001
	OFF- ON^**^	Score	10 (7, 14)	9 (6, 12)	10 (8, 14)	0.010
S&E	OFF	Score	80 (70, 90)	90 (80, 90)	70 (60, 80)	< 0.001
	ON^**^	Score	90 (80, 90)	90 (90, 100)	90 (80, 90)	< 0.001
	ON- OFF^**^	Score	10 (0, 20)	10 (0, 10)	10 (10, 20)	< 0.001
DRS-2	Total^***^	Raw score	124 (112, 131)	128 (122, 134)	121 (106, 129)	< 0.001

ADO, age at disease onset; DA, dopamine agonist; DD, disease duration; DRS-2, dementia rating scale-2; Laterality-laterality of motor symptoms at disease onset; LED-levodopa equivalent dose; S&E, Schwab and England Activities of Daily Living Scale; UPDRS, unified Parkinson's disease rating scale. ^*^Ropinirole was the only DA taken by 106/114 patients (93%). ^**^UPDRS-II, UPDRS-III and S&E in ON state was limited to patients medicated with antiparkinsonian medication (n = 276 patients). ^***^DRS-2 was only applied to patients with at least 3 years of education (n = 255). However, 2 were unable to complete the task. Data are presented as frequencies (%) and medians (Q1, Q3). Chi-square test and Mann-Whitney test were used for group comparison.

The genotypic frequencies for IL-1β rs16944 (*n* = 276) were 111 CC (40.2%), 133 CT (48.2%), and 32 TT (11.6%); for DRD3 rs6280 (*n* = 281) were 125 TT (44.5%), 112 TC (39.9%), and 44 CC (15.7%); and for GRIN2B rs7301328 were 115 CC (40.9%), 129 CG (45.9%), and 37 GG (13.2%). The IL-1β rs16944 genotype frequency did not differ (p=0.261) between patients with (43.3% CC, 44.9% CT, 11.8% TT) and without (33.7% CC, 55.1% CT, 11.2% TT) a caregiver/informant present at the time of the assessment. The IL-1β rs16944 C allele was present in 244 (88.4%) and the T allele in 165 (59.8%) of patients. The DRD3 rs6280 T allele was present in 237 (84.3%) and the C allele in 156 (55.5%) of patients. The GRIN2B rs7301328 was present in 244 (86.8%) and the G allele in 166 (59.1%) of patients. In previous studies from our center, Hardy–Weinberg equilibrium (HWE) was evaluated in a healthy control population, which represents the standard approach in genetic association studies, and all three genes were in HWE. In our PD cohort, genotype frequencies for DRD3 showed a modest deviation from HWE (*p* = 0.03), whereas the other analyzed genes remained consistent with HWE (IL-1β rs16944: *p* = 0.408; GRIN2B rs7301328: *p* = 0.931).

### 3.2 Impulse control behaviors and disorders

ICBDs since PD onset (QUIP-Anytime) were recorded in 63 (22%) PD patients; 44 (16%) had just ICDs (i.e., compulsive gambling, buying, sex, and/or eating behaviors); 15 (5%) had more than 1 ICD; 27 (10%) had related behaviors (i.e., hobbyism and/or punding); and 8 (3%) had DDS. Among patients taking dopamine agonist (*n* = 114), ICBDs were documented in 37 (33%) patients, ICDs in 25 (22%), hobbyism-punding in 20 (18%), and DDS in 4 (4%). The presence of ICBDs were related to male sex, younger age, higher education, younger age at disease onset, right onset of motor symptoms, longer disease duration, higher levodopa equivalent dose, dopamine agonist (93% were taking ropinirole), more severe symptoms in motor activities of daily living as measured by UPDRS-II in OFF, poorer functional independence as measured by S&E in OFF, and greater absolute gains on the UPDRS-II, UPDRS-III and S&E with dopamine replacement therapy.

The presence of ICBDs (frequency per genotype: 18% CC, 21% CT, 44% TT, *p* = 0.008, Cramer's V=0.188) and ICDs (frequency per genotype: 13% CC, 14% CT, and 34% TT, *p* = 0.008, Cramer's *V* = 0.188) were related to rs16944 genotype (Table 2).

**Table 2:** Genotypical associations with neuropsychiatric measures.

Neuropsychiatric symptoms		Total frequency	Frequency x Genotype				
			IL-1B rs16944				
QUIP-anytime		*n* = 281	CC = 111	CT = 133	TT = 32	*P*	*Cramer's V*
	ICBDs	63 (22%)	20 (18%)	28 (21%)	14 (44%)	0.008	0.188
	ICDs	44 (16%)	14 (13%)	18 (14%)	11 (34%)	0.008	0.188
NPI		*n* = 191	CC = 81	CT = 84	TT = 21	*P*	*Eta^2^ or Cramer's V*
	Total score in logarithmic scale	2.30 (1.4, 3.2)	2.2 (1.4, 3.1)	2.2 (1.4, 3.2)	3.0 (2.2, 3.6)	0.044	0.023
	Hallucinations	39 (21%)	11 (14%)	20 (24%)	8 (38%)	0.033	0.191
			DRD3 rs6280				
HADS		*n* = 246	TT = 115	TC = 92	CC = 39	*P*	*Cramer's V*
	Anxiety ≥11	45 (18%)	27 (24%)	9 (10%)	9 (23%)	0.028	0.170

HADS, hospital anxiety and depression scale; ICBDs, impulse control and related behaviors disorders; ICDs, impulse control disorders; NPI, neuropsychiatric inventory; QUIP-Anytime—Questionnaire for Impulsive-Compulsive Disorders in Parkinson's Disease—Anytime Short. NPI was applied to caregivers/informants of 191 PD patients, however only 186 had IL1B rs16944 genotyping. The NPI Total Score was converted into natural logarithmic scale. For the purpose of the conversion, a NPI total raw score of zero was replaced by one. NPI item Hallucinations was marked “not applicable” for 1 patient and it was treated as missing data. The HADS was only applied to PD patients with ≥3 years of education (n = 255). However, HADS was discontinued in 9 patients, due to the patients' inability to complete the task. Data are presented as frequencies (%) and medians (Q1, Q3). Chi-square test and Kruskal-Wallis test were used for group comparisons. *Post-hoc* analyses were applied to calculate effect sizes.

Subsequent simple logistic regression analyses revealed that ICBDs (OR = 3.54, 95%CI: 1.51, 8.28, *p* = 0.004) and ICDs (OR = 3.63, 95%CI: 1.45, 9.11, *p* = 0.006) were more frequent in patients with rs16944 TT genotype than CC genotype. These associations between rs16944 TT genotype and ICBDs and ICDs remained statistically significant when demographic and clinical variables were taken into consideration (Table 3).

**Table 3:** Predictors of impulse-control and related behavioral disorders (ICBDs) and impulse control disorders (ICDs) in PD (*n* = 281).

Genetic, Demographic and clinical variables		QUIP-anytime with ICBDs									QUIP-Anytime with ICDs								
		Simple logistic regressions			Multiple logistic regression						Simple logistic regressions			Multiple logistic regression					
					Model 1			Model 2						Model 3			Model 4		
		OR	95%CI	*P*	Adj OR	95%CI	*P*	Adj OR	95%CI	*P*	OR	95%CI	*P*	Adj OR	95%CI	*P*	Adj OR	95%CI	*P*
rs16944	CC (*n* = 111)																		
	CT (*n* = 133)	1.21	0.64, 2.30	0.553	1.66	0.80, 3.45	0.173	1.66	0.80, 3.44	0.177	1.08	0.51, 2.29	0.832	1.38	0.62, 3.10	0.432	1.35	0.60, 3.03	0.469
	TT (*n* = 32)	3.54	1.51, 8.28	0.004	5.78	2.08, 16.06	< 0.001	6.32	2.25, 17.74	< 0.001	3.63	1.45, 9.11	0.006	5.26	1.92, 14.37	0.001	4.85	7.73, 13.63	0.003
Sex	Male (*n* = 146)	2.63	1.45, 4.80	0.002	2.89	1.42, 5.87	0.003	3.00	1.48, 6.09	0.002	1.98	1.01, 3.89	0.046	2.24	1.06, 4.72	0.034	2.21	1.04, 4.68	0.039
Age	Years	0.95	0.93, 0.98	< 0.001	0.94	0.91, 0.96	< 0.001	0.94	0.91, 0.97	< 0.001	0.95	0.93, 0.98	< 0.001	0.95	0.92, 0.98	< 0.001	0.94	0.91, 0.98	< 0.001
ADO	Years	0.93	0.91, 0.96	< 0.001							0.94	0.91, 0.96	< 0.001						
Laterality	Right (*n* = 154)	1.89	1.05, 3.41	0.033	2.30	1.16, 4.58	0.018	2.44	1.21, 4.92	0.012	1.73	0.88, 3.40	0.110						
DD	Years	1.10	1.05, 1.16	< 0.001	1.11	1.05, 1.18	< 0.001	1.12	1.05, 1.18	< 0.001	1.09	1.04, 1.15	< 0.001	1.11	1.05, 1.17	< 0.001	1.11	1.05, 1.17	< 0.001
LED	Mg	1.001	1.000, 1.001	< 0.001	1.001	1.000, 1.001	0.096	-	-	-	1.001	1.000, 1.001	0.015	-	-	-	-	-	-
DA	Yes (*n* = 114)	2.61	1.47, 4.62	0.001	-	-	-	-	-	-	2.19	1.14, 4.20	0.019	-	-	-	-	-	-
UPDRS-II UPDRS-III	OFF	1.06	1.02, 1.10	0.002	-	-	-	-	-	-	1.05	1.01, 1.09	0.022	-	-	-	-	-	-
	ON	1.03	0.98, 1.07	0.287							1.04	0.99, 1.10	0.129						
	OFF-ON	1.10	1.04, 1.17	< 0.001				-	-	-	1.06	0.997, 1.13	0.060						
	OFF	1.02	0.99, 1.04	0.158							1.01	0.99, 1.04	0.302						
	ON	0.99	0.96, 1.02	0.451							1.00	0.96, 1.03	0.827						
	OFF-ON	1.10	1.05, 1.16	< 0.001				1.06	0.995, 1.12	0.074	1.07	1.07, 1.13	0.017				-	-	-
S& E	OFF	0.98	0.97, 0.998	0.025	-	-	-	-	-	-	0.99	0.97, 1.01	0.287						
	ON	1.00	0.98, 1.02	0.805							0.99	0.97, 1.02	0.487						
	ON-OFF	1.04	1.02, 1.07	0.001				-	-	-	1.03	0.98, 1.04	0.374						

ADO, age at disease onset; DA, dopamine agonist; DD, disease duration; Laterality, laterality of motor symptoms at disease onset; LED, levodopa equivalent dose; QUIP-Anytime—Questionnaire for Impulsive-Compulsive Disorders in Parkinson's Disease—Anytime Short; S&E—Schwab and England Activities of Daily Living Scale; UPDRS—Unified Parkinson's Disease Rating Scale. Higher UPDRS-II OFF-ON, UPDRS-III OFF-ON and S&E ON-OFF indicate greater absolute gains on the specific scale with dopamine replacement therapy. In the total sample (*n* = 281), ICBDs and ICDs were present in 63 and 44 patients, respectively. IL1B rs16944 genotyping was only performed in 276 individuals (62 with ICBDs and 42 with ICDs). One of the patients without rs16944 genotype had ICBDs and ICDs. ON state assessments were not available in 5 patients, who were not medicated. For QUIP-Anytime with ICBDs, two multivariable logistic regression models were considered: Model 1 included rs16944 polymorphism, sex, age, laterality, DD, LED, DA, UPDRS-II OFF, and S&E OFF; and Model 2 included rs16944 polymorphism, sex, age, laterality, DD, LED, DA, UPDRS-II OFF, UPDRS-II OFF-ON, UPDRS-III OFF-ON, S&E OFF, and S&E ON-OFF. For QUIP-Anytime with ICDs, two multivariable logistic regression models were considered: Model 3 included rs16944 polymorphism, sex, age, DD, LED, DA, and UPDRS-II OFF; and Model 4 included rs16944 polymorphism, sex, age, DD, LED, DA, UPDRS-II OFF, and UPDRS-III OFF-ON. The backward likelihood ratio selection method was applied with a removal criterion of *p* > 0.100. The removed variables are marked with (-).

The frequencies of ICBDs (22.0 vs. 23.3%, *p* = 0.801) and ICDs (15.7 vs. 15.6%, *p* = 0.974) were compared between patients with (*n* = 191) and without (*n* = 90) a caregiver/informant present at the time of the assessment and no differences were found. Multiple logistic regression analyses did not reveal significant rs16944 genotype x presence of a caregiver/informant interaction effects on the occurrence of ICBDs or ICDs.

Noteworthy, patients with IL-1β rs16944 TT genotype had similar demographic and clinical characteristics to the CC genotype. However, CT genotype had shorter disease duration than those with CC genotype; they also had smaller gains on the UPDRS-II OFF-ON and on S&E OFF-ON than those with CC and TT genotypes; and tended to have lower UPDRS-II OFF and higher S&E OFF (Supplementary Table 4).

Patients with the IL-1β rs16944 wild-type allele (i.e., C allele was present in 244/276 patients) had less ICBDs (43.8 vs. 19.7%, *p* = 0.002, Phi = −0.185) and ICDs (34.4 vs. 13.1%, *p* = 0.004, Phi = −0.188; Supplementary Table 3), whereas patients with the GRIN2B rs7301328 variant G allele (i.e., present in 166/281 patients) had less hobbyism-punding (13.9 vs. 6.6%, *p* = 0.042, Phi = −0.122; Supplementary Table 2). These associations remained significant when demographic and clinical variables were considered as covariates. No other associations (*p* > 0.05) were found between ICBDs and the studied polymorphisms (Supplementary Tables 1–3).

### 3.3 Caregiver/informant-reported neuropsychiatric symptoms

NPI was available only in PD patients with caregiver/informant present at the time of the assessment (*n* = 191). Patients with NPI had older age at assessment and at disease onset, fewer years of education, longer disease duration, higher levodopa equivalent dose, less dopamine agonist, more severe motor symptoms (UPDRS-II and UPDRS-III in OFF and ON states), lower functional independence (S&E in OFF and ON states), and poorer cognitive performance (DRS-2) (Table 1).

The median NPI total raw score was 10 (Q1 = 4, Q3 = 24) and the median in the natural logarithmic scale was 2.30 (Q1 = 1.4, Q3 = 3.2). Depression/dysphoria was the most frequently reported neuropsychiatric symptom (72%) within the previous month, followed by anxiety (50%), apathy/indifference (46%), irritability/lability (37%), agitation/aggression (29%), sleep and nighttime behavior disorders (28%), hallucinations in (21%), appetite and eating disorders (21%), aberrant motor behavior (14%), disinhibition (14%), delusions (9%), and elation/euphoria (5%).

NPI total scores (in natural logarithmic scale) were related to higher UPDRS-II (OFF and ON states), higher UPDRS-III (OFF and ON states) and lower S&E (OFF and ON states). In addition to the associations with more severe motor symptoms (UPDRS-II and UPDRS-III in OFF and ON states) and with lower functional independence (S&E in OFF and ON), hallucinations were related to longer disease duration and greater absolute gains in the S&E with antiparkinsonian medication (Table 4).

**Table 4:** Predictors of NPI Total score and hallucinations in PD (*n* = 191): simple and multiple linear and logistic regressions.

Genetic, Demographic and clinical variables		NPI total score (logarithmic scale)^*^									NPI with hallucinations^**^								
		Simple linear regressions			Multiple linear regression						Simple logistic regressions			Multiple logistic regression					
					Model 1			Model 2						Model 3			Model 4		
		*ß*	95%CI	*P*	Adj *ß*	95%CI	*P*	Adj *ß*	95%CI	*P*	OR	95%CI	*P*	Adj OR	95%CI	*P*	Adj OR	95%CI	*P*
IL1B rs16944	CC (*n* = 81)	−0.25	−0.60, 2.56	0.152															
	CT (*n* = 84)	−0.01	−0.36, 0.34	0.945							1.99	0.89, 4.47	0.096	0.94	0.94, 5.35	0.068	2.26	0.94, 5.47	0.070
	TT (*n* = 22)	0.63	0.10, 1.16	0.020	0.52	0.02, 1.03	0.043	0.50	0.001, 0.99	0.049	3.92	1.32, 11.60	0.014	3.74	1.17, 11.91	0.026	4.01	1.20, 13.39	0.024
Sex	Male (*n* = 99)	−0.00	−0.34, 0.34	0.993							0.87	0.43, 1.75	0.689						
Age	Years	0.01	−0.01, 0.02	0.441							1.01	0.97, 1.05	0.641						
ADO	Years	0.00	−0.02, 0.02	0.952							0.98	0.95, 1.01	0.272						
Laterality	Right (*n* = 105)	0.01	−0.33, 0.35	0.945							1.21	0.59, 2.47	0.601						
DD	Years	0.02	−0.01, 0.05	0.205							1.09	1.03, 1.16	0.004	-	-	-	-	-	-
LED	Mg	0.00	0.00, 0.00	0.314							1.001	1.000, 1.001	0.073						
DA	Yes (*n* = 68)	−0.35	−0.70, 0.00	0.052							0.65	0.30, 1.40	0.270						
UPDRS-II	OFF	0.05	0.03, 0.07	< 0.001	0.05	0.03, 0.07	< 0.001				1.13	1.07, 1.19	< 0.001	1.13	1.07, 1.19	< 0.001	1.09	1.02, 1.16	0.012
	ON	0.07	0.04, 0.09	< 0.001				0.06	0.03, 0.09	< 0.001	1.13	1.07, 1.20	< 0.001				-	-	-
	OFF-ON	0.01	−0.02, 0.05	0.524							1.06	0.99, 1.13	0.085						
UPDRS-III	OFF	0.02	0.01, 0.04	0.002	-	-	-	−0.04	−0.08, −0.01	0.011	1.08	1.04, 1.12	< 0.001	-	-	-	-	-	-
	ON	0.04	0.02, 0.05	< 0.001				0.05	0.02, 0.08	0.002	1.09	1.05, 1.14	< 0.001				1.05	1.00, 1.10	0.047
	OFF-ON	−0.01	−0.04, 0.02	0.484							1.03	0.97, 1.09	0.347						
S& E	OFF	−0.02	−0.03, −0.01	< 0.001	-	-	-	-	-	-	0.96	0.94, 0.98	< 0.001	-	-	-	-	-	-
	ON	−0.03	−0.04, −0.02	< 0.001				-	-	-	0.96	0.93, 0.98	< 0.001				-	-	-
ON-OFF	0.00	−0.01, 0.02	0.639							1.04	1.01, 1.07	0.008						

ADO, age at disease onset; DA, dopamine agonist; DD, disease duration; Laterality, laterality of motor symptoms at disease onset; LED, levodopa equivalent dose; NPI, neuropsychiatric inventory; S&E, Schwab and England activities of daily living scale; UPDRS, unified Parkinson's disease rating scale. Higher UPDRS-II OFF-ON, UPDRS-III OFF-ON and S&E ON-OFF indicate greater absolute gains on the specific scale with dopamine replacement therapy. ^*^NPI Total Scores were converted into natural logarithmic scale. For the purpose of the conversion, a NPI total raw score of zero was replaced by one. ^**^Hallucinations were present in 39 patients, absent in 151 patients, and was marked “not applicable” for 1 patient (which was treated as missing data). IL1B rs16944 genotyping was only performed in 187 patients and none of the patients without rs16944 genotype had hallucinations. ON state assessments were not available in 1 patient, who was not medicated. Two multivariable linear regression models were considered for NPI Total score: Model 1 included rs16944 polymorphism, UPDRS-II OFF, UPDRS-III OFF, and S&E OFF; and Model 2 included rs16944 polymorphism, UPDRS-II OFF, UPDRS-II ON, UPDRS-III OFF, UPDRS-III ON, S&E OFF, and S&E ON. For NPI Hallucinations, two multivariable logistic regression models were considered: Model 3 included rs16944 polymorphism, DD, UPDRS-II OFF, UPDRS-III OFF, and S&E OFF; and Model 4 included rs16944 polymorphism, DD, UPDRS-II OFF, UPDRS-II ON, UPDRS-III OFF, UPDRS-III ON, S&E OFF, and S&E ON. A stepwise selection approach was applied to the multiple linear regression analyses (i.e., backward selection) and to the multiple logistic regression analyses (i.e., backward likelihood ratio). The removal criterion was set at *p* > 0.100. The removed variables are marked with (−).

**Figure 1:**
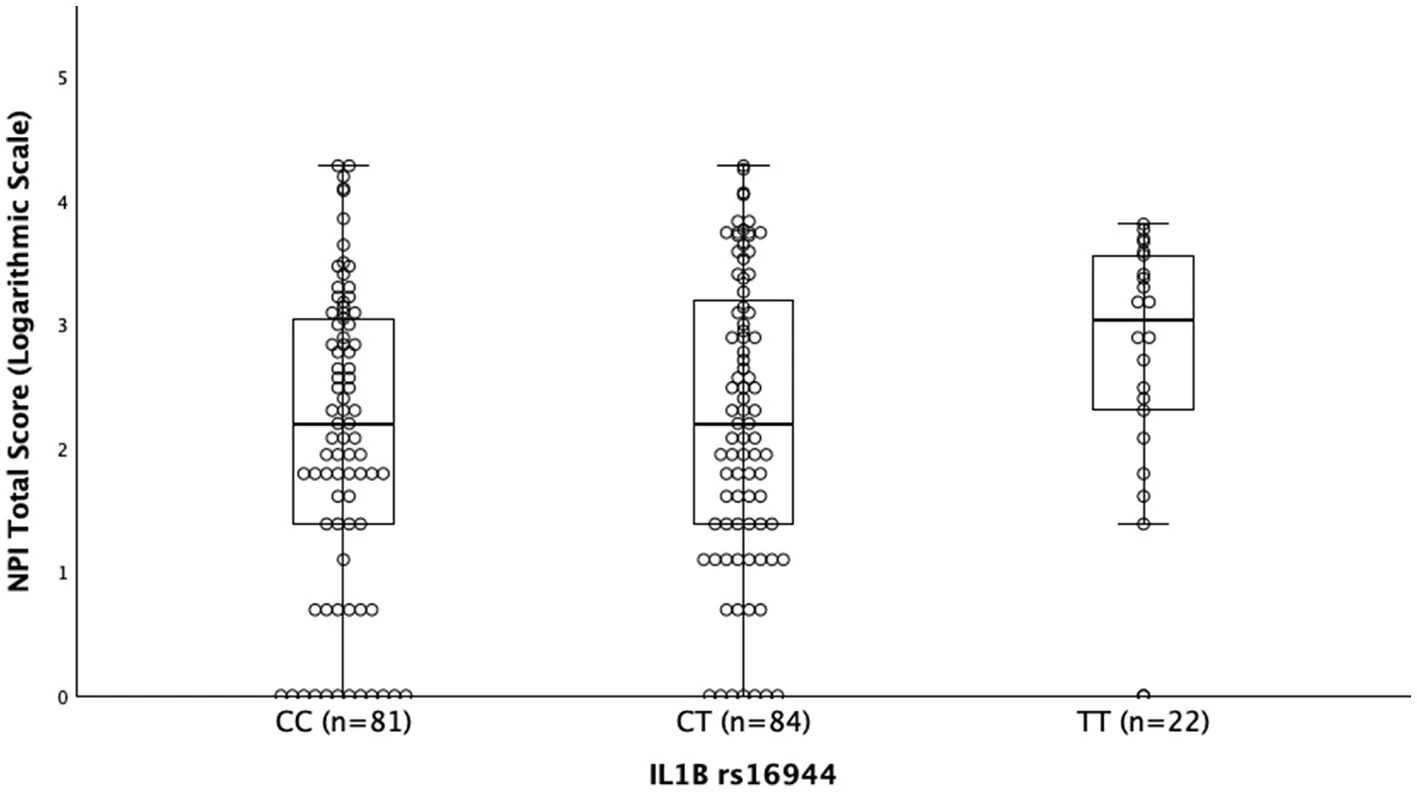
Boxplot of neuropsychiatric inventory (NPI) total scores according to IL1B rs16944 genotype. NPI total scores were converted to natural logarithmic scale. For the purpose of the conversion, a NPI total raw score of zero was replaced by one.

The NPI total score varied significantly according to IL-1β rs16944 genotype (median total scores in logarithmic scale: CC = 2.2, CT = 2.2, TT = 3.0, *p* = 0.044) (Table 2 and Figure 1). NPI total score (in natural logarithmic scale) was related to IL-1β rs16944 TT genotype (ß = 0.63, 95%CI: 0.10, 1.16, *p* = 0.020). Reports of hallucinations on the NPI were also significantly related with rs16944 genotype (CC = 14%, CT = 24%, TT = 38%, *p* = 0.033), with TT patients having a higher odds of hallucinations (OR = 3.92, 95%CI: 1.32, 11.60, *p* = 0.014) (Tables 2, 4). Both associations between NPI and rs16944 TT genotype remained statistically significant when clinical predictors were taken into consideration (Table 4). Noteworthy, patients with rs16944 CT genotype also tended to have more hallucinations than patients with CC genotype, especially after adjusting for clinical variables (Table 4).

Regarding associations with the studied alleles, patients with the IL-1β rs16944 wild-type C allele had fewer neuropsychiatric symptoms (median NPI Total scores in natural logarithmic scale: 2.20 vs. 3.03, *p* = 0.015; Eta^2^ = 0.031); patients with the IL-1β rs16944 variant T allele had more hallucinations (14 vs. 27%, *p* = 0.030, Phi = 0.159) and agitation/aggression (21 vs. 35%, *p* = 0.037, Phi = 0.152); and patients with GRIN2B rs7301328 variant G allele had more depression/dysphoria (64 vs. 78%, *p* = 0.026, Phi = 0.161) on the NPI (Supplementary Tables 2, 3). When significant demographic and clinical predictors were taken into consideration, the IL-1β rs16944 variant T remained statistically associated with more hallucinations and agitation/aggression; the GRIN2B rs7301328 variant G allele remained associated with more depression/dysphoria; and the IL-1β rs16944 wild-type C allele remained related to lower NPI total scores, but not with presence of hallucinations (Table 5).

**Table 5:** Allelic associations with neuropsychiatric measures.

Neuropsychiatric measures	Polymorphism	Number of patients with the allele	Regression analyses						
QUIP-anytime			Simple logistic regression			Multiple logistic regression			
			OR	95%CI	*P*	Regression	Adj OR	95%CI	*P*
ICBDs (*n* = 62/276)	IL1B rs16944	Allele C (*n* = 244/276)	0.32	0.15, 0.68	0.003	Model 1	0.21	0.09, 0.53	< 0.001
ICDs (*n* = 43/276)	IL1B rs16944	Allele C (*n* = 244/276)	0.29	0.13, 0.65	0.003	Model 2	0.23	0.09, 0.56	0.001
Hobbyism-Punding (*n* = 27/281)	GRIN2B rs7301328	Allele G (*n* = 166/281)	0.44	0.20, 0.99	0.046	Model 3	0.33	0.13, 0.81	0.015
NPI			Simple linear regression			Multiple linear regression			
			*ß*	95%CI	*P*		Adj *ß*	95%CI	*P*
Total score in logarithmic scale	IL1B rs16944	Allele C (*n* = 165/186)	−0.63	−0.16, −0.10	0.020	Model 4	−0.52	−1.03, −0.02	0.043
			Simple logistic regression			Multiple logistic regression			
NPI			OR	95%CI	*P*		Adj OR	95%CI	*P*
Hallucinations (*n* = 39/186)	IL1B rs16944	Allele C (*n* = 165/186)	0.38	0.14, 0.99	0.047	Model 5	-	-	-
Hallucinations (*n* = 39/186)	IL1B rs16944	Allele T (*n* = 105/186)	2.31	1.07, 4.99	0.032	Model 5	2.54	1.12, 5.79	0.027
Agitation/Aggression (*n* = 54/187)	IL1B rs16944	Allele T (*n* = 106/186)	2.02	1.04, 3.94	0.039	Model 6	2.09	1.07, 4.11	0.032
Depression/Dysphoria (*n* = 138/191)	GRIN2B rs7301328	Allele G (*n* = 111/191)	2.06	1.09, 3.92	0.027	Model 7	2.09	1.09, 4.00	0.026
HADS			Simple logistic regression			Multiple logistic regression			
			OR	95%CI	*P*		Adj OR	95%CI	*P*
Anxiety ≥11 (*n* = 45/246)	DRD3 rs6280	Allele C (*n* = 131/246)	0.52	0.27, 1.00	0.051	Model 8	0.47	0.23, 0.94	0.033

HADS, hospital anxiety and depression scale; ICBDs, impulse control and related behaviors disorders; ICDs, impulse control disorders; NPI, neuropsychiatric inventory; QUIP-Anytime—Questionnaire for Impulsive-Compulsive Disorders in Parkinson's Disease—Anytime Short. The NPI Total Score was converted into natural logarithmic scale. For the purpose of the conversion, a NPI total raw score of zero was replaced by one. NPI item Hallucinations was marked “not applicable” for 1 patient and it was treated as missing data. The HADS was only applied to PD patients with ≥3 years of education (n = 255). However, HADS was discontinued in 9 patients, due to the patients' inability to complete the task. The multiple regression models were developed based on univariate analyses results. Only genetic, demographic, and clinical variables with significant association (p < 0.05) with the neuropsychiatric measure were considered. Furthermore, no ON state neurological assessments were taken into account. The initial regression models were: Model 1—**allele, sex, age, laterality (laterality of motor symptoms at disease onset), disease duration (DD), levodopa equivalent dose (LED)**, dopamine agonist (DA), Unified Parkinson's Disease Rating Scale (UPDRS) II OFF, and Schwab and England Activities of Daily Living Scale (S&E) OFF; Model 2—**allele, sex, age, DD**, LED, DA, UPDRS-II OFF, and S&E OFF; Model 3—**allele, sex, age, DD**, LED, and DA; Model 4—**allele, UPDRS-II OFF**, UPDRS-III OFF, and S&E OFF; Model 5—allele, DD, **UPDRS-II OFF**, UPDRS-III OFF, and S&E OFF; Model 6—**allele** and **sex**; Model 7—**allele** and **UPDRS-II OFF**; and Model 8—**allele, age**, DD, **UPDRS-II OFF**, UPDRS-III OFF, and S&E OFF. Backward selection and backward likelihood ration selection (when appropriate) applied, with a removal criterion of p > 0.100. The variables remaining in the final regression models are written in bold.

No other associations (*p* > 0.05) were found between NPI and the studied polymorphisms (Supplementary Tables 1–3).

### 3.4 Self-reported anxiety and depression

Among patients with at least 3 years of education (*n* = 255), only 246 were able to complete HADS. Those unable to answer the HADS questionnaire (*n* = 9) had older age (medians: 76 vs. 68, *p* = 0.018), lower education (median number of years: both 4, *p* = 0.008), poorer cognitive performance (only 7/9 without HADS were able to complete DRS-2; median DRS-2 total scores: 105 vs. 125, *p* = 0.022), and more severe motor symptoms in OFF and ON (UPDRS-III OFF scores: 47 vs. 31, *p* < 0.001; UPDRS-III ON scores: 37 vs. 20, *p* = 0.007), though, no significant differences were observed regarding age at disease onset (*p* = 0.293), disease duration (*p* = 0.242) or levodopa equivalent dose (*p* = 0.240). Patients unable to complete HADS had more delusions (57 vs. 7%, *p* = 0.001) and hallucinations (71 vs. 16%, *p* = 0.003) and tended to have more neuropsychiatric symptoms (median NPI total scores: 27 vs. 9, *p* = 0.060) reported by caregivers / informants, but not ICBDs (*p* > 0.999). Similar differences were observed when comparing all those that did not complete HADS (i.e., due to education restrictions and/or inability to complete the task) and those that responded the questionnaire.

The median HADS total score was 14 (Q1 = 10, Q3 = 19). The frequency of anxiety (HADS anxiety≥11) was 18% and frequency of depression (HADS depression≥11) was 21%. As previously reported, the frequency of anxiety varied with DRD3 rs6280 genotype (23.5% TT, 9.8% TC, 23.1% CC, *p* = 0.028, Cramer's *V* = 0.170). DRD3 rs6280 TC genotype had less anxiety symptoms (OR = 0.35, 95%CI: 0.16, 0.80, *p* = 0.012) than patients with TT genotype. Patients with the rs6280 C allele had slightly less anxiety (23.5 vs. 13.7%, *p* = 0.049, Phi = −0.126). This association with the C allele remained statistically significant when controlling for demographic and clinical predictors.

No other significant association was found between HADS and the studied polymorphisms (Supplementary Tables 1–3).

## 4 Discussion

Study results suggest that IL-1β rs16944 TT genotype is a genetic susceptibility factor for the development of neuropsychiatric symptoms PD, including impulse control and related disorders (ICBDs and ICDs), total neuropsychiatric symptom burden and hallucinations, even when other significant predictors (including demographic and clinical variables, both during OFF and ON medication periods) were considered. These significant association with the IL-1β rs16944 TT, but not with the CT genotype, suggest a possible recessive effect. However, a threshold effect is more likely, especially for hallucinations, which tended to be more frequent in patients with CT than CC even though patients with rs16944 CC genotype had longer disease duration than those with CT genotype. Furthermore, the rs16944 T allele was associated with more reports of hallucinations and of agitation/aggression in PD patients, even when other significant predictors were taken into account.

A previous study by (Redensek, 2019) found no significant association between rs16944 genotype and ICDs and visual hallucinations in PD. Differences in cohort characteristics (e.g., disease duration, medication exposure, and ethnicity) and assessment procedures (e.g., the use of standard screening questionnaires for neuropsychiatric symptoms vs. the recording of symptoms based on a structured interview and a review of medical records) could explain the inconsistent findings. Noteworthy, Redenšeke et al.' reported ORs of the rs16944 TT genotype for ICDs and visual hallucinations are within the 95%CI from our study, suggesting a general tendency.

It is known that neuroinflammatory processes play an important role in PD pathogenesis (Tansey et al., 2022). The rs16944 T allele is generally associated with higher secretion of IL-1β and that homozygous carriers of the rs16944 T allele have an increased risk of developing PD (Schulte et al., 2002; Wahner et al., 2007). Our findings extend this evidence by suggesting that this functional polymorphism may also be involved in the occurrence of certain neuropsychiatric symptoms in PD.

ICDs and visual hallucinations are common complications of dopamine-replacing medications in PD (Debove et al., 2024; Lizarraga et al., 2020). Though, they may also stem from the disease itself or the combination of the disease and the treatment (Hejazi, 2020). If left untreated, ICDs and psychotic symptoms can have a devastating impact on the patient's life (Chaudhuri and Schapira, 2009). The underlying pathophysiological mechanisms of ICDs and hallucinations in PD have not been fully established and the role of neuroinflammation is still unclear.

Consistent with the hypothesis of a hypodopaminergic vulnerability in PD patients with ICDs in the context of hyperdopaminergic state with dopamine replacement therapy (Theis et al., 2021), in our cohort the discrepancy between OFF and ON states in motor and non-motor symptoms and in activities of daily living was greater in PD patients with ICBDs. There is evidence that increased inflammation may exacerbate dysfunction within the dopaminergic reward system by disrupting dopamine synthesis, storage, and receptor signaling (Felger and Miller, 2012; Theis et al., 2021). However, this interaction is complex and is yet incompletely understood.

In our cohort, ICDs were documented in 16% of patients and 6% had more than one ICD, which is consistent with the 14 and 4% respectively documented by the DOMINION multicentre study with 3090 PD patients (Weintraub et al., 2010). In accordance with the literature, male sex, younger age at assessment and at disease onset, longer disease duration, higher levodopa equivalent dose, and dopamine agonist use were risk factors for the occurrence of ICBDs/ICDs (Weintraub et al., 2010; Weintraub, 2008). In the present study, the association between current dopamine agonist use and history of ICDs since PD onset was relatively modest, likely due to medication adjustment when ICDs occurred. In our cohort, PD patients with right side disease onset had an increased risk of having ICBDs, but not specifically with ICDs. Most studies in the literature have reported negative findings regarding a potential effect of laterality of motor symptoms at PD onset on the occurrence of ICDs (Barone et al., 2019; Bastiaens et al., 2013; Premi et al., 2016). Though, it has been suggested that ICDs in PD are associated with a left-hemispheric dopaminergic system imbalance (Premi et al., 2016).

In the present study, based on caregivers/informants' responses to NPI, 21% of PD patients had hallucinations in the previous month. This frequency of hallucinations was based on a subset of the total cohort (i.e., only 68% of patients had caregiver assessments), who had older age, longer disease duration, poorer cognition, and greater disease severity than those without caregiver data (Table 1). Studies in other cohorts have reported prevalence rates for complex visual hallucinations ranging from 22 to 38% (Fenelon and Alves, 2010). Consistent with our finding that visual hallucinations are more common in PD patients who are homozygous for the IL-1β rs16944 T allele, it has been reported that this allele is associated with increased transition to psychosis in non-PD individuals with ultra-high risk of psychosis (Mostaid et al., 2019). Another genetic study, found that a gene polymorphism (i.e., rs1800795) related to reduced expression levels of a pro-inflammatory cytokine (i.e., IL-6) was associated with lower risk of visual hallucinations in PD patients (Redenšek et al., 2020). Non-genetic studies have also provided evidence of a relationship between low-grade inflammation and the occurrence of hallucinations or illusions in PD (Sawada et al., 2014) and in non-PD clinical populations, namely, schizophrenia (Orlovska-Waast et al., 2019). Furthermore, autoimmune central nervous system conditions frequently present with psychosis (Pollak et al., 2020).

In PD, dopamine replacement therapies may exacerbate hallucinations in vulnerable patients, such as those with low ventral striatal dopamine transporter density (Jaakkola et al., 2017). However, unlike ICBDs, dopaminergic treatments may not be the primary driving factor in the occurrence of hallucinations in PD. Converging evidence points to cholinergic degeneration, which lowers perceptual thresholds, and to synergistic interactions between cholinergic deficits and dopaminergic fluctuations playing a pivotal role in the occurrence of visual hallucinations in PD (Hatsuta et al., 2025; Ignatavicius et al., 2025; Liu et al., 2026; Stahl, 2016). Abnormalities in serotonin 2A receptor neurotransmission (i.e., upregulation of 5HT2A receptors in the prefrontal and visual/temporal cortex areas) and disruptions to the noradrenergic and GABAergic systems have also been linked to visual hallucinations in PD (Ballanger et al., 2010; Huot et al., 2010; Ignatavicius et al., 2025; Stahl, 2016; Powell et al., 2020). Clinicopathological studies have revealed correlations between visual hallucinations and Lewy body pathology in neocortical and limbic regions of PD patients and the presence of Alzheimer's disease pathology further increased the risk of visual hallucinations in PD (Jacobson et al., 2014).

In our cohort, caregivers/informants' reports of agitation/aggression were more frequent in patients with IL-1β rs16944 variant allele. This finding supports the notion that IL-1β is involved in aggressive behavior. Non-PD studies that have explored the neurochemistry of aggression, both in animals and in humans, have consistently found positive correlations between aggressiveness and circulating IL-1β levels in cerebrospinal fluid and serum (Coccaro et al., 2015; Hassanain et al., 2003; Pesce et al., 2013).

Neuropsychiatric manifestations in PD are heterogeneous multifactorial phenomena with complex and diverse underlying pathophysiological mechanisms (Marras and Chaudhuri, 2016; Sauerbier et al., 2016). Our observation that IL-1β rs16944 relates more strongly to ICDs and selected NPI domains (i.e., ICDs, hallucinations, and agitation/aggression) may reflect a specific neuropsychiatric-inflammatory profile, which may cluster with other non-motor symptoms(Marras and Chaudhuri, 2016; Sauerbier et al., 2016). Future studies may benefit from integrating subtype-informed modeling (e.g., motor and non-motor clustering frameworks) to test whether inflammation-related genetic variation preferentially maps onto particular neuropsychiatric trajectories.

In our cohort, no significant association was found between a screening measure of anxiety and depression (HADS) and IL-1β rs16944 polymorphism. However, this analysis did not include patients with less than 3 years of education nor patients who were unable to answer the self-report questionnaire. These restrictions lead to the exclusion of older patients with more severe disease, which constitute a relevant bias and a generalizability limitation. Noteworthy, there is evidence in the literature that anxiety, in particular state anxiety, and depression are related to inflammation in PD (Ramanzini et al., 2022; Zhou et al., 2025).

As a secondary study goal, the effects of DRD3 rs6280 and GRIN2B rs7301328 genotypes on neuropsychiatric symptoms were explored. Neither polymorphism was related to ICBDs or ICDs in our cohort. The literature has provided mixed findings regarding potential associations between DRD3 rs6280 and GRIN2B rs7301328 genotypes and the occurrence of ICBDs and ICDs in PD (Hall et al., 2021; Krishnamoorthy et al., 2016; Lee et al., 2009; Zainal Abidin et al., 2015). In our cohort of PD patients, GRIN2B rs7301328 variant carriers had more frequent depressive symptoms, which aligns with evidence linking GRIN2B variants to mood disorders in non-PD populations (Zhang et al., 2014; Zhao et al., 2011). As previously described, DRD3 rs6280 heterozygous had fewer anxiety symptoms (Gonçalves et al., 2024).

This is a single center study conducted in a predominantly Caucasian cohort (99.6%) from the northern part of Portugal, which limits the generalizability of the findings to the broader PD population. Moreover, some key study findings (from the NPI data) were derived from a subset of patients, who were older and had more severe disease. Likewise, the HADS analyses excluded patients with lower educational attainment and more advanced cognitive impairment. Thus, introducing potential selection biases into the analyses of NPI and HADS and further limiting the generalizability of the findings.

In the present study, multiple comparisons were performed between neuropsychiatric symptoms and polymorphisms, which increased the risk of type I error. The *post-hoc* analyses revealed that the effect sizes were weak/small and that the FDR were greater than 5%. Therefore, the significant associations found in this study should be interpreted with caution and ought to be viewed as hypothesis-generating findings. Future studies with independent validation cohorts are warranted to confirm the present findings.

Our hospital-based outpatient cohort was recruited consecutively and included patients in all stages of the disease, including non-medicated patients and bedridden patients. Another strength of the study is the concomitant assessment of motor (in OFF and ON states by movement disorders specialists) and non-motor (by a neuropsychologist) symptoms, which were performed blinded to the genetic results. The standard questionnaires chosen to explore neuropsychiatric symptoms in PD are some of the most widely used screening instruments in both research and clinical practice (Weintraub et al., 2022). These screening instruments are very useful tools to identify potential cases, but do not capture the full clinical spectrum of each neuropsychiatric disorder in PD and are not sufficient to make a definitive diagnosis (Martinez-Martin et al., 2016). Future studies ought to use more precise neuropsychiatric phenotyping when exploring the relationship between neuropsychiatric symptoms and genetic risk factors in PD.

In conclusion, study results suggest that IL-1β rs16944 TT genotype confers vulnerability to certain neuropsychiatric symptoms in PD, particularly ICDs, hallucinations, and agitation/aggression. This genotype has been previously linked to high-production of the pro-inflammatory cytokine IL-1β. However, the present observational study did not measure circulating IL-1β nor explored indicators of inflammation. Future studies ought to combine pro-inflammatory cytokine genotypes, direct measurements of circulating pro-inflammatory cytokines, biomarkers of systemic inflammation and neuroinflammation, and neuroimaging measures. A longitudinal follow-up would also be helpful to clarify the relationship between inflammation, dopamine dysregulation, and neuropsychiatric manifestations in PD.

## Data Availability

The datasets presented in this study can be found in online repositories. The names of the repository/repositories and accession number(s) can be found below: https://sigarra.up.pt, the raw data supporting the conclusions of this article will be made available by the authors upon justified request, in compliance with ethical standards and data protection regulations.
