# Supplementary material for: Inflammation and neuropsychiatric symptoms in Parkinson's disease: a genetic association
Source: Front Aging Neurosci. 2026 Aug 31;18:1877657. doi: 10.3389/fnagi.2026.1877657 (PMC13572645; doi:10.3389/fnagi.2026.1877657)
Supplement: Supplementary file 1 [file Data_Sheet_1.docx]

Supplementary Table 1: Neuropsychiatric measures. Associations with IL1B rs16944, DRD3 rs6280, and GRIN2B rs7301328 genotypes

|  |  |  | Total |  | IL1B rs16944 | | | |  | DRD3 rs6280 | | | |  | GRIN2B rs7301328 | | | |
| --- | --- | --- | --- | --- | --- | --- | --- | --- | --- | --- | --- | --- | --- | --- | --- | --- | --- | --- |
| QUIP-anytime |  |  | n=281 |  | CC=111 | CT=133 | TT=32 | *P* |  | TT=125 | TC=112 | CC=44 | *p* |  | CC=115 | CG=129 | GG=37 | *P* |
|  | ICBDs | Positive | 63 (22%) |  | 20 (18%) | 28 (21%) | 14 (44%) | **0.008** |  | 29 (23%) | 23 (21%) | 11 (25%) | 0.802 |  | 29 (25%) | 30 (23%) | 4 (11%) | 0.179 |
|  | ICDs | Positive | 44 (16%) |  | 14 (13%) | 18 (14%) | 11 (34%) | **0.008** |  | 21 (17%) | 18 (16%) | 5 (11%) | 0.686 |  | 17 (15%) | 24 (19%) | 3 (8%) | 0.285 |
|  | Hobbyism -Punding | Positive | 27 (10%) |  | 8 (7%) | 14 (11%) | 5 (16%) | 0.340 |  | 12 (10%) | 8 (7%) | 7 (16%) | 0.247 |  | 16 (14%) | 10 (8%) | 1 (3%) | 0.082 |
|  | Dopamine Dysregulation Syndrome | Positive | 8 (3%) |  | 6 (5%) | 2 (2%) | 0 (0%) | 0.113 |  | 2 (2%) | 5 (5%) | 1 (2%) | 0.404 |  | 3 (3%) | 4 (3%) | 1 (3%) | 0.972 |
|  |  |  |  |  |  |  |  |  |  |  |  |  |  |  |  |  |  |  |
| NPI* |  |  | n=191 |  | CC=81 | CT=84 | TT=22 | *P* |  | TT=81 | TC=79 | CC=31 | *P* |  | CC=80 | CG=87 | GG=24 | *P* |
|  | Total (Frequency x Severity) | Score in  logarithmic scale | 2.30 (1.39 , 3.18) |  | 2.20 (1.39 , 3.07) | 2.20 (1.39 , 3.23) | 3.03 (2.25 , 3.56) | **0.044** |  | 2.48 (1.61 , 3.30) | 2.08 (1.10 , 3.09) | 2.20 (1.39 , 3.40) | 0.199 |  | 1.95 (1.44 , 3.12) | 2.49 (1.39 , 3.22) | 2.83 (1.66 , 3.30) | 0.283 |
|  | Delusions | Positive | 17 (9%) |  | 6 (7%) | 8 (10%) | 3 (14%) | 0.655 |  | 10 (12%) | 3 (4%) | 4 (13%) | 0.114 |  | 8 (10%) | 9 (10%) | 0 (0%) | 0.261 |
|  | Hallucinations | Positive | 39 (21%) |  | 11 (14%) | 20 (24%) | 8 (38%) | **0.033** |  | 21 (26%) | 10 (13%) | 8 (26%) | 0.077 |  | 14 (18%) | 20 (23%) | 5 (21%) | 0.703 |
|  | Agitation/Aggression | Positive | 55 (29%) |  | 17 (21%) | 28 (33%) | 9 (41%) | 0.090 |  | 18 (22%) | 25 (32%) | 12 (39%) | 0.173 |  | 26 (33%) | 22 (25%) | 7 (29%) | 0.589 |
|  | Depression/Dysphoria | Positive | 138 (72%) |  | 59 (73%) | 58 (69%) | 18 (82%) | 0.485 |  | 61 (75%) | 54 (68%) | 23 (74%) | 0.596 |  | 51 (64%) | 68 (78%) | 18 (79%) | 0.083 |
|  | Anxiety | Positive | 96 (50%) |  | 38 (47%) | 41 (49%) | 14 (64%) | 0.370 |  | 47 (58%) | 35 (44%) | 14 (45%) | 0.183 |  | 38 (48%) | 44 (51%) | 14 (58%) | 0.646 |
|  | Elation/Euphoria | Positive | 10 (5%) |  | 5 (6%) | 4 (5%) | 1 (5%) | 0.908 |  | 5 (6%) | 4 (5%) | 1 (3%) | 0.819 |  | 3 (4%) | 5 (6%) | 2 (8%) | 0.649 |
|  | Apathy/Indifference | Positive | 86 (45%) |  | 38 (47%) | 36 (43%) | 11 (50%) | 0.786 |  | 42 (52%) | 29 (37%) | 15 (48%) | 0.165 |  | 30 (38%) | 42 (48%) | 14 (61%) | 0.104 |
|  | Disinhibition | Positive | 26 (14%) |  | 10 (13%) | 11 (13%) | 5 (23%) | 0.449 |  | 15 (19%) | 6 (8%) | 5 (16%) | 0.112 |  | 10 (13%) | 14 (16%) | 2 (8%) | 0.558 |
|  | Irritability/Lability | Positive | 71 (37%) |  | 24 (30%) | 34 (41%) | 11 (50%) | 0.141 |  | 34 (42%) | 23 (29%) | 14 (45%) | 0.145 |  | 28 (35%) | 32 (37%) | 11 (46%) | 0.626 |
|  | Aberrant motor behavior | Positive | 27 (14%) |  | 10 (12%) | 14 (17%) | 3 (14%) | 0.728 |  | 9 (11%) | 11 (14%) | 7 (23%) | 0.296 |  | 9 (11%) | 14 (16%) | 4 (17%) | 0.622 |
|  | Sleep and Nighttime Behavior Disorders | Positive | 51 (28%) |  | 21 (27%) | 24 (30%) | 5 (24%) | 0.799 |  | 24 (32%) | 20 (26%) | 7 (23%) | 0.633 |  | 22 (29%) | 24 (29%) | 5 (22%) | 0.769 |
|  | Appetite and Eating Disorders | Positive | 40 (21%) |  | 14 (17%) | 18 (22%) | 6 (27%) | 0.547 |  | 15 (19%) | 19 (24%) | 6 (19%) | 0.692 |  | 16 (20%) | 19 (22%) | 5 (21%) | 0.969 |
|  |  |  |  |  |  |  |  |  |  |  |  |  |  |  |  |  |  |  |
| HADS** |  |  | n=246 |  | CC=94 | CT=121 | TT=27 | *P* |  | TT=115 | TC=92 | CC=39 | *P* |  | CC=101 | CG=111 | GG=34 | *p* |
|  | Anxiety | $\geq$11 | 45 (18%) |  | 19 (20%) | 22 (18%) | 3 (11%) | 0.558 |  | 27 (24%) | 9 (10%) | 9 (23%) | **0.028** |  | 21 (21%) | 21 (19%) | 3 (9%) | 0.288 |
|  | Depression | $\geq$11 | 51 (21%) |  | 16 (17%) | 30 (25%) | 5 (19%) | 0.360 |  | 21 (18%) | 18 (20%) | 12 (31%) | 0.235 |  | 20 (20%) | 23 (21%) | 8 (24%) | 0.898 |

*The Neuropsychiatric Inventory (NPI) was applied to 191 caregivers / informants. NPI item Hallucinations was marked “not applicable” for 1 patient and it was treated as missing data. **The Hospital Anxiety and Depression Scale (HADS) was only applied to PD patients with $\geq$3 years of education (n=255). However, HADS was discontinued in 9 patients, due to the patient’s inability to complete the task. The rs16944 genotype was only performed in 187 patients. Data are presented as frequencies (%) and medians (Q1 , Q3). Chi-square test and Kruskal-Wallis test were used for group comparisons.
