# Supplementary material for: Inflammation and neuropsychiatric symptoms in Parkinson's disease: a genetic association
Source: Front Aging Neurosci. 2026 Aug 31;18:1877657. doi: 10.3389/fnagi.2026.1877657 (PMC13572645; doi:10.3389/fnagi.2026.1877657)
Supplement: Supplementary file 2 [file Data_Sheet_2.docx]

Supplementary Table 2: Neuropsychiatric measures. Associations with IL1B rs16944, DRD3 rs6280, and GRIN2B rs7301328 variant alleles

|  |  |  |  | IL1B rs16944 | | |  | DRD3 rs6280 | | |  | GRIN2B rs7301328 | | |
| --- | --- | --- | --- | --- | --- | --- | --- | --- | --- | --- | --- | --- | --- | --- |
|  |  |  |  | Without T (n=111) | With T (n=165) | *p* |  | Without C (n=125) | With C (n=156) | *P* |  | Without G (n=115) | With G (n=166) | *P* |
| QUIP | ICBDs | Positive |  | 20 (18.0%) | 42 (25.5%) | 0.147 |  | 29 (23.2%) | 34 (21.8%) | 0.779 |  | 29 (25.2%) | 34 (20.5%) | 0.349 |
|  | ICDs | Positive |  | 14 (12.6%) | 29 (17.6%) | 0.265 |  | 21 (16.8%) | 23 (14.7%) | 0.637 |  | 17 (14.8%) | 27 (16.3%) | 0.737 |
|  | Hobbyism -Punding | Positive |  | 8 (7.2%) | 19 (11.5%) | 0.237 |  | 12 (9.6%) | 15 (9.6%) | 0.997 |  | 16 (13.9%) | 11 (6.6%) | **0.042** |
|  | Dopamine Dysregulation Syndrome | Positive |  | 6 (5.4%) | 2 (1.2%) | 0.064 |  | 2 (1.6%) | 6 (3.8%) | 0.306 |  | 3 (2.6%) | 5 (3.0%) | >0.999 |
|  |  |  |  |  |  |  |  |  |  |  |  |  |  |  |
|  |  |  |  | Without T (n=81) | With T (n=106) | *p* |  | Without C (n=81) | With C (n=110) | *P* |  | Without G (n=80) | With G (n=111) | *P* |
| NPI | Total (Frequency x Severity) | Score in logarithmic scale |  | 2.20 (1.39 , 3.07) | 2.40 (1.39 , 3.38) | 0.182 |  | 2.49 (1.61 , 3.30) | 2.20 (1.39 , 3.15) | 0.162 |  | 1.95 (1.44 , 3.12) | 2.56 (1.39 , 3.26) | 0.150 |
|  | Delusions | Positive |  | 6 (7.4%) | 11 (10.4%) | 0.484 |  | 10 (12.3%) | 7 (6.4%) | 0.151 |  | 8 (10.0%) | 9 (8.1%) | 0.651 |
|  | Hallucinations | Positive |  | 11 (13.6%) | 28 (26.7%) | **0.030** |  | 21 (26.3%) | 18 (16.4%) | 0.096 |  | 14 (17.7%) | 25 (22.5%) | 0.419 |
|  | Agitation/Aggression | Positive |  | 17 (21.0%) | 37 (34.9%) | **0.037** |  | 18 (22.2%) | 37 (33.6%) | 0.085 |  | 26 (32.5%) | 29 (26.1%) | 0.337 |
|  | Depression/Dysphoria | Positive |  | 59 (72.8%) | 76 (71.7%) | 0.863 |  | 61 (75.3%) | 77 (70.0%) | 0.418 |  | 51 (63.7%) | 87 (78.4%) | **0.026** |
|  | Anxiety | Positive |  | 38 (46.9%) | 55 (51.9%) | 0.500 |  | 47 (58.0%) | 49 (44.5%) | 0.066 |  | 38 (47.5%) | 58 (52.3%) | 0.517 |
|  | Elation/Euphoria | Positive |  | 5 (6.2%) | 5 (4.7%) | 0.748 |  | 5 (6.2%) | 5 (4.5%) | 0.746 |  | 3 (3.8%) | 7 (6.3%) | 0.525 |
|  | Apathy/Indifference | Positive |  | 38 (46.9%) | 47 (44.3%) | 0.726 |  | 42 (51.9%) | 44 (40.4%) | 0.116 |  | 30 (37.5%) | 56 (50.9%) | 0.067 |
|  | Disinhibition | Positive |  | 10 (12.5%) | 16 (15.1%) | 0.613 |  | 15 (18.8%) | 11 (10.0%) | 0.083 |  | 10 (12.5%) | 16 (14.5%) | 0.685 |
|  | Irritability/Lability | Positive |  | 24 (29.6%) | 45 (42.5%) | 0.072 |  | 34 (42.0%) | 37 (33.6%) | 0.239 |  | 28 (35.0%) | 43 (38.7%) | 0.598 |
|  | Aberrant motor behavior | Positive |  | 10 (12.3%) | 17 (16.0%) | 0.477 |  | 9 (11.1%) | 18 (16.4%) | 0.303 |  | 9 (11.3%) | 18 (16.2%) | 0.331 |
|  | Sleep and Nighttime Behavior Disorders | Positive |  | 21 (26.9%) | 29 (29.0%) | 0.760 |  | 24 (31.6%) | 27 (25.5%) | 0.366 |  | 22 (28.6%) | 29 (27.6%) | 0.888 |
|  | Appetite and Eating Disorders | Positive |  | 14 (17.3%) | 24 (22.9%) | 0.350 |  | 15 (18.80%) | 25 (22.7%) | 0.507 |  | 16 (20.3%) | 24 (21.6%) | 0.820 |
|  |  |  |  |  |  |  |  |  |  |  |  |  |  |  |
|  |  |  |  | Without T (n=94) | With T (n=194) | *p* |  | Without C (n=115) | With C (n=131) | *P* |  | Without G (n=101) | With G (n=145) | *P* |
| HADS | Anxiety | $\geq$11 |  | 19 (20.2%) | 25 (16.9%) | 0.514 |  | 27 (23.5%) | 18 (13.7%) | **0.049** |  | 21 (20.8%) | 24 (16.6%) | 0.397 |
|  | Depression | $\geq$11 |  | 16 (17.0%) | 35 (23.6%) | 0.218 |  | 21 (18.3%) | 30 (22.9%) | 0.370 |  | 20 (19.8%) | 31 (21.4%) | 0.764 |

*The Neuropsychiatric Inventory (NPI) was applied to 191 caregivers / informants. NPI item Hallucinations was marked “not applicable” for 1 patient and it was treated as missing data. **The Hospital Anxiety and Depression Scale (HADS) was only applied to PD patients with $\geq$3 years of education (n=255). However, HADS was discontinued in 9 patients, due to the patient’s inability to complete the task. The rs16944 genotype was only performed in 187 patients. Data are presented as frequencies (%) and medians (Q1 , Q3). Chi-square test and Mann-Whitney test were used for group comparisons.
