# Supplementary material for: Inflammation and neuropsychiatric symptoms in Parkinson's disease: a genetic association
Source: Front Aging Neurosci. 2026 Aug 31;18:1877657. doi: 10.3389/fnagi.2026.1877657 (PMC13572645; doi:10.3389/fnagi.2026.1877657)
Supplement: Supplementary file 3 [file Data_Sheet_3.docx]

Supplementary Table 3: Neuropsychiatric measures. Associations with IL1B rs16944, DRD3 rs6280, and GRIN2B rs7301328 wild-type alleles

|  |  |  |  | IL1B rs16944 | | |  | DRD3 rs6280 | | |  | GRIN2B rs7301328 | | |
| --- | --- | --- | --- | --- | --- | --- | --- | --- | --- | --- | --- | --- | --- | --- |
|  |  |  |  | Without C (n=32) | With C (n=244) | *p* |  | Without T (n=44) | With T (n=237) | *P* |  | Without C (n=37) | With C (n=244) | *P* |
| QUIP | ICBDs | Positive |  | 14 (43.8%) | 48 (19.7%) | **0.002** |  | 11 (25.0%) | 52 (21.9%) | 0.655 |  | 4 (10.8%) | 59 (24.2%) | 0.069 |
|  | ICDs | Positive |  | 11 (34.4%) | 32 (13.1%) | **0.004** |  | 5 (11.4%) | 39 (16.5%) | 0.393 |  | 3 (8.1%) | 41 (16.8%) | 0.175 |
|  | Hobbyism -Punding | Positive |  | 5 (15.6%) | 22 (9.0%) | 0.218 |  | 7 (15.9%) | 20 (8.4%) | 0.159 |  | 1 (2.7%) | 26 (10.7%) | 0.225 |
|  | Dopamine Dysregulation Syndrome | Positive |  | 0 (0.0%) | 8 (3.3%) | 0.602 |  | 1 (2.3%) | 7 (3.0%) | >0.999 |  | 1 (2.7%) | 7 (2.9%) | >0.999 |
|  |  |  |  |  |  |  |  |  |  |  |  |  |  |  |
|  |  |  |  | Without C (n=22) | With C (n=165) | *P* |  | Without T (n=31) | With T (n=160) | *P* |  | Without C (n=24) | With C (n=167) | *P* |
| NPI | Total (Frequency x Severity) | Score in logarithmic scale |  | 3.03 (2.25 , 3.56) | 2.20 (1.39 , 3.09) | **0.015** |  | 2.20 (1.39 , 3.40) | 2.30 (1.39) | 0.606 |  | 2.83 (1.66 , 3.30) | 2.20 (1.39 , 3.14) | 0.273 |
|  | Delusions | Positive |  | 3 (13.6%) | 14 (8.5%) | 0.429 |  | 4 (12.9%) | 13 (8.1%) | 0.487 |  | 0 (0.0%) | 17 (10.2%) | 0.136 |
|  | Hallucinations | Positive |  | 8 (38.1%) | 31 (18.8%) | 0.050 |  | 8 (25.8%) | 31 (19.5%) | 0.426 |  | 5 (20.8%) | 34 (20.5%) | >0.999 |
|  | Agitation/Aggression | Positive |  | 9 (40.9%) | 45 (27.3%) | 0.185 |  | 12 (38.7%) | 43 (26.9%) | 0.183 |  | 7 (29.2%) | 48 (28.7%) | 0.966 |
|  | Depression/Dysphoria | Positive |  | 18 (81.8%) | 117 (70.9%) | 0.283 |  | 23 (74.2%) | 115 (71.9%) | 0.792 |  | 19 (79.2%) | 119 (71.3%) | 0.418 |
|  | Anxiety | Positive |  | 14 (63.6%) | 79 (47.9%) | 0.165 |  | 14 (45.2%) | 82 (51.2%) | 0.535 |  | 14 (58.3%) | 82 (49.1%) | 0.398 |
|  | Elation/Euphoria | Positive |  | 1 (4.5%) | 9 (5.5%) | >0.999 |  | 1 (3.2%) | 9 (5.6%) | >0.999 |  | 2 (8.3%) | 8 (4.8%) | 0.365 |
|  | Apathy/Indifference | Positive |  | 11 (50.0%) | 74 (44.8%) | 0.649 |  | 15 (48.4%) | 71 (44.7%) | 0.702 |  | 14 (60.9%) | 72 (43.1%) | 0.109 |
|  | Disinhibition | Positive |  | 5 (22.7%) | 21 (12.8%) | 0.202 |  | 5 (16.1%) | 21 (13.2%) | 0.775 |  | 2 (8.3%) | 24 (14.5%) | 0.540 |
|  | Irritability/Lability | Positive |  | 11 (50.0%) | 58 (35.2%) | 0.175 |  | 14 (45.2%) | 57 (35.6%) | 0.315 |  | 11 (45.8%) | 60 (35.9%) | 0.348 |
|  | Aberrant motor behavior | Positive |  | 3 (13.6%) | 24 (14.5%) | >0.999 |  | 7 (22.6%) | 20 (12.5%) | 0.160 |  | 4 (16.7%) | 23 (13.8%) | 0.754 |
|  | Sleep and Nighttime Behavior Disorders | Positive |  | 5 (23.8%) | 45 (28.7%) | 0.642 |  | 7 (23.3%) | 44 (28.9%) | 0.532 |  | 5 (21.7%) | 46 (28.9%) | 0.473 |
|  | Appetite and Eating Disorders | Positive |  | 6 (27.3%) | 32 (19.5%) | 0.403 |  | 6 (19.4%) | 34 (21.4%) | 0.800 |  | 5 (20.8%) | 35 (21.1%) | 0.978 |
|  |  |  |  |  |  |  |  |  |  |  |  |  |  |  |
|  |  |  |  | Without C (n=27) | With C (n=215) | *P* |  | Without T (n=39) | With T (n=207) | *P* |  | Without C (n=34) | With C (n=212) | *P* |
| HADS | Anxiety | $\geq$11 |  | 3 (11.1%) | 41 (19.1%) | 0.431 |  | 9 (23.1%) | 36 (17.4%) | 0.400 |  | 3 (8.8%) | 42 (19.8%) | 0.124 |
|  | Depression | $\geq$11 |  | 5 (18.5%) | 46 (21.4%) | 0.730 |  | 12 (30.8%) | 39 (18.8%) | 0.092 |  | 8 (23.5%) | 43 (20.3%) | 0.665 |

*The Neuropsychiatric Inventory (NPI) was applied to 191 caregivers / informants. NPI item Hallucinations was marked “not applicable” for 1 patient and it was treated as missing data. **The Hospital Anxiety and Depression Scale (HADS) was only applied to PD patients with $\geq$3 years of education (n=255). However, HADS was discontinued in 9 patients, due to the patient’s inability to complete the task. The rs16944 genotype was only performed in 187 patients. Data are presented as frequencies (%) and medians (Q1 , Q3). Chi-square test and Mann-Whitney test were used for group comparisons.
