# Supplementary material for: Inflammation and neuropsychiatric symptoms in Parkinson's disease: a genetic association
Source: Front Aging Neurosci. 2026 Aug 31;18:1877657. doi: 10.3389/fnagi.2026.1877657 (PMC13572645; doi:10.3389/fnagi.2026.1877657)
Supplement: Supplementary file 4 [file Data_Sheet_4.docx]

Supplementary Table 4: Demographical and clinical characterization according to IL-1β rs16944 genotype.

|  | | | IL-1β rs16944 in Total Sample (n=276) | | |  |  |  | IL-1β rs16944 in Subgroup with NPI (n=187) | | |  |  |
| --- | --- | --- | --- | --- | --- | --- | --- | --- | --- | --- | --- | --- | --- |
|  | | | CC (n=111) | CT (n=133) | TT (n=32) | *p* | *Post-Hoc* |  | CC (n=81) | CT (n=84) | TT (n=22) | *p* | *Post-Hoc* |
| Sex | | Male | 62 (56%) | 68 (51%) | 15 (47%) | 0.604 |  |  | 44 (54%) | 44 (52%) | 10 (46%) | 0.761 |  |
| Age | | Years | 69 (60 , 78) | 70 (63 , 77) | 70 (64 , 75) | 0.721 |  |  | 69 (61 , 79) | 73 (67 , 78) | 73 (67 , 76) | 0.490 |  |
| Education | | Years | 4 (4 , 9) | 4 (4 , 6) | 4 (4 , 6) | 0.946 |  |  | 4 (4 , 5) | 4 (4 , 5) | 4 (4 , 5) | 0.915 |  |
| Age at disease onset | | Years | 60 (51 , 67) | 63 (55 , 70) | 61 (54 , 69) | 0.182 |  |  | 60 (51 , 69) | 66 (58 , 70) | 61 (53 , 70) | 0.081 |  |
| Laterality | | Right | 111 (56%) | 71 (53%) | 16 (50%) | 0.827 |  |  | 47 (58%) | 44 (52%) | 10 (46%) | 0.532 |  |
| DD | | Years | 7 (5 , 12) | 6 (4 , 10) | 9 (5 , 11) | **0.032** | **CT<CC; p=0.047**  CT-TT; p=0.277  CC-TT; p>0.999 |  | 9 (5 , 14) | 7 (4 , 10) | 11 (8 , 13) | **0.015** | **CT<CC; p=0.034**  CT-TT; p=0.092  CC-TT; p>0.999 |
| LED | | Mg | 900 (480 , 1200) | 640 (400 , 1070) | 885 (400 , 1230) | 0.152 |  |  | 970 (570 , 1310) | 800 (500 , 1280) | 1034 (585 , 1336) | 0.320 |  |
| DA* | | Yes | 111 (46%) | 48 (36%) | 13 (41%) | 0.296 |  |  | 35 (43%) | 24 (29%) | 8 (36%) | 0.146 |  |
| UPDRS-II | OFF | Score | 14 (9 , 21) | 12 (8 , 19) | 16 (10 , 24) | 0.076 |  |  | 16 (12 , 22) | 16 (10 , 23) | 19 (15 , 25) | 0.327 |  |
|  | ON** | Score | 8 (4 , 11) | 7 (4 , 12) | 9 (3 , 13) | 0.777 |  |  | 8 (5 , 12) | 9 (6 , 14) | 10 (4 , 15) | 0.585 |  |
|  | OFF-ON** | Score | 6 (4 , 9) | 5 (2 , 8) | 8 (5 , 10) | **0.004** | **CT<CC; p=0.022**  **CT<TT; p=0.028**  CC-TT; p=0.383 |  | 7 (4 , 10) | 6 (3 , 9) | 8 (6 , 13) | 0.120 |  |
| UPDRS-III | OFF | Score | 34 (27 , 42) | 30 (23 , 39) | 31 (25 , 40) | 0.151 |  |  | 36 (30 , 43) | 36 (29 , 44) | 34 (26 , 47) | 0.870 |  |
|  | ON** | Score | 23 (16 , 28) | 21 (15 , 27) | 19 (16 , 30) | 0.591 |  |  | 24 (17 , 30) | 25 (18 , 30) | 20 (16 , 34) | 0.919 |  |
|  | OFF-ON** | Score | 10 (8 , 14) | 10 (7 , 13) | 11 (6 , 14) | 0.324 |  |  | 11 (8 , 15) | 10 (7 , 14) | 11 (8 , 17) | 0.590 |  |
| S&E | OFF | Score | 80 (60 , 90) | 80 (70 , 90) | 70 (70 , 80) | 0.068 |  |  | 70 (60 , 80) | 75 (60 , 90) | 70 (58 , 80) | 0.310 |  |
|  | ON** | Score | 90 (80 , 90) | 90 (80 , 90) | 90 (80 , 90) | 0.888 |  |  | 90 (80 , 90) | 90 (80 , 90) | 90 (80 , 90) | 0.920 |  |
|  | ON-OFF** | Score | 10 (0 , 20) | 10 (0 , 10) | 10 (10 , 20) | **0.004** | **CT<CC; p=0.044**  **CT<TT; p=0.013**  CC-TT; p=0.599 |  | 10 (10 , 20) | 10 (0 , 20) | 20 (10 , 20) | **0.048** | CT-CC; p=0.171  CT-TT; p=0.110  CC-TT; p=0.599 |
| DRS-2 | Total*** | Raw score | 126 (110 , 132) | 124 (112 , 130) | 121 (116 , 130) | 0.927 |  |  | 120 (107 , 131) | 123 (105 , 128) | 120 (109 , 129) | 0.785 |  |

ADO - age at disease onset; DA - dopamine agonist; DD - disease duration; DRS-2 - Dementia Rating Scale-2; Laterality-laterality of motor symptoms at disease onset; LED-levodopa equivalent dose; S&E - Schwab and England Activities of Daily Living Scale; UPDRS - Unified Parkinson’s Disease Rating Scale. *Ropinirole was the only DA taken by 106/114 patients (93%). **UPDRS-II, UPDRS-III and S&E in ON state was limited to patients medicated with antiparkinsonian medication (n=276 patients). ***DRS-2 was only applied to patients with at least 3 years of education (n=255). However, 2 were unable to complete the task. Data are presented as frequencies (%) and medians (Q1 , Q3). Chi-square test and Mann-Whitney test were used for group comparison. In the Post-hoc analyses, pairwise comparisons adjusted by the Bonferroni correction for multiple comparisons was applied.
